# The biology of how circumcision reduces HIV susceptibility: broader implications for the prevention field

**DOI:** 10.1186/s12981-017-0167-6

**Published:** 2017-09-12

**Authors:** Jessica L. Prodger, Rupert Kaul

**Affiliations:** 1 Department of Epidemiology, Johns Hopkins Bloomberg School of Public Health, Baltimore, USA; 2 Department of Medicine, University of Toronto, Medical Sciences Building, Room #6356, 1 King’s College Circle, Toronto, ON M5S 1A8 Canada

**Keywords:** HIV-1, T-cells, Chemokines, Microbiome, Circumcision, Foreskin

## Abstract

Circumcision reduces heterosexual HIV-1 acquisition in men by at least 60%. However, the biological mechanisms by which circumcision is protective remain incompletely understood. We test the hypothesis that the sub-preputial microenvironment created by the foreskin drives immune activation in adjacent foreskin tissues, facilitating HIV-1 infection through a combination of epithelial barrier disruption, enhanced dendritic cell maturation, and the recruitment/activation of neutrophils and susceptible CD4 T cell subsets such as Th17 cells. Furthermore, we provide evidence that the genital microbiome may be an important driver of this immune activation. This suggests that new modalities to reduce genital immune activation and/or alter the genital microbiome, used alone or in combination with topical microbicides, may be of significant benefit to HIV prevention.

Male circumcision reduces HIV-1 (HIV) infection in heterosexual men by approximately 60% [1]. However, the biological mechanism by which circumcision confers this protection remains poorly understood. The foreskin constitutes a fold of skin that covers the coronal sulcus, glans, and urethral meatus of the non-erect penis, and the distal aspect of the penile shaft on the erect penis. While the foreskin is one continuous sheet of skin, the portion of the foreskin that lies against the glans on the non-erect penis is referred to as the “inner” foreskin, while that which is exposed to the air at all times is referred to as the “outer” foreskin. The folding of the foreskin on the non-erect penis creates a sub-preputial space between the inner foreskin and glans that is largely anaerobic, and which is eliminated on the erect penis. By removing the foreskin surgically, circumcision permanently eliminates the sub-preputial space and exposes the glans to air on both the erect and non-erect penis.

Early speculation as to the protective biological mechanism of circumcision assumed that the relatively sheltered inner foreskin had a thinner layer of keratin (stratum corneum) than the outer foreskin and the penile shaft. Keratin is an insoluble layer of non-viable cells that limits the diffusion of HIV into the underlying living tissue, reducing access to HIV-susceptible cells [2], and so removing tissue with a thinner keratin layer would plausibly protect against HIV. However, subsequent studies quantifying the depth of stratum corneum have not found consistent differences between the inner and outer foreskin [3–7], and so this is unlikely to be the mechanism.

An alternative hypothesis is that elimination of the sub-preputial space reduces HIV susceptibility by altering the local immune environment of the penile skin. Productive infection after sexual exposure to HIV is relatively rare, with significant heterogeneity in susceptibility between individuals [8]. Additionally, despite the swarm of virus quasispecies that is present in an infected partner’s genital secretions, only a single viral strain establishes systemic infection [9]. This suggests that the establishment of productive infection in genital tissue constitutes a considerable barrier to the virus, and data from our group and others demonstrate that the local genital immune milieu is a major component of this barrier.

Models of SIV transmission in the female genital tract show that infection begins with the establishment of a productive focus of infected CD4 T-cells that expands through local viral replication, followed by systemic dissemination after several days [10]. Genital immune activation may facilitate this process through several mechanisms. The first is through a reduction in the barrier function of genital tissue. In men, virus is able to enter foreskin tissue both through passive diffusion across the epithelium [2] and through active transport by migrating dendritic cells, which can transfer infectious virions to dermal CD4 T-cells [11]. Initial penetration of virions across the stratum corneum is inefficient [2], but local inflammation in the skin disrupts the epithelial barrier through tissue remodeling, with increased HIV penetration at areas of decreased gap junction proteins [2]. Similarly, vaginal inflammation in women is associated with decreased epidermal cell differentiation and cornified envelope pathways [12], which may reduce barrier integrity and enhance virus penetration.

Inflammation also has important effects on genital immune cells that may enhance HIV acquisition. Not only do local inflammatory signals enhance dendritic cell migration and increase *trans* infection of T-cells [13, 14], but immune activation also increases both the number and HIV-permissiveness of genital CD4 T-cells. This would be expected to stochastically increase the likelihood of productive infection after HIV exposure and, in keeping with this, the number of mucosal CCR5/CD4+ T-cells is a key determinant of macaque susceptibility after rectal SIV challenge [15], and larger foreskin size is associated with increased risk of HIV acquisition in adult men [16]. Activated CD4 T-cells are also more permissive to infection and produce more virus than resting cells, with specific CD4 T-cell subsets being preferential HIV targets [17]. Not only do Th17 cells express high levels of CCR5 and demonstrate enhanced in vitro HIV susceptibility [18, 19], but this subset comprises almost two-thirds of early SIV-infected cells in the genital tract of female macaques [20], despite the relative rarity of Th17 cells in genital tissue (<20% of CD4 T-cells [18, 20, 21]). Further evidence that Th17 cells are central to genital HIV susceptibility comes from individuals who are Highly Exposed to HIV but remain SeroNegative (HESN): HESN men have a reduced relative abundance of Th17 cells in their foreskin tissues [22], and HESN women demonstrate reduced genital expression of Th17 cytokines [23].

Longitudinal studies clearly confirm the importance of genital immune activation in HIV susceptibility. Uncircumcised men who acquired HIV were more likely to have had preceding high levels of the chemokines IL-8 and Monokine Induced by interferon-gamma (MIG) in their sub-preputial space than those who remained uninfected [24]. IL-8 is produced by epithelial and other cells and is best known for its recruitment of neutrophils, which provide an important defense against extracellular pathogens partially through the recruitment of Th17 cells [25, 26], and IL-8 concentrations in the sub-preputial space correlated with the density of both neutrophils and Th17 cells in foreskin tissue [24]. Furthermore, men who acquired HIV had higher preceding levels of innate anti-microbial proteins in their sub-preputial space, particularly of neutrophil-derived α-defensins [27]. While some of these innate molecules have anti-HIV activity in vitro, they also act as pro-inflammatory signaling molecules, promoting epithelial remodeling and inflammation, which may overshadow any anti-HIV activity [28, 29].

Based on these data, our overall hypothesis is that circumcision reduces HIV susceptibility by reducing local inflammation in penile tissues, preventing loss of barrier integrity and reducing HIV-target cell density in exposed skin. While it is not feasible to prove this by obtaining paired skin biopsies from a man’s penis pre- and post-circumcision, coronal sulcus IL-8 levels progressively decline for at least 2 years after circumcision [24]. In addition, several studies have compared immune cells between the inner and outer aspects of foreskin tissue, under the assumption that the latter would resemble the penile shaft skin that remains after circumcision. The inner foreskin harbors an increased density of CD4 T-cells [2, 7] and releases increased levels of pro-inflammatory cytokines [7, 30]. In situ explant studies show that dendritic cells from the inner foreskin demonstrate increased environmental sampling [31] and are better able to transfer infectious HIV to dermal T-cells [32], a characteristic of dendritic cells that have been matured through exposure to bacterial antigens [13, 14, 30]. Therefore it seems that tissues adjacent to the sub-preputial space (i.e. the inner foreskin) display a pro-inflammatory environment that is more conducive to HIV, and that circumcision eliminates the sub-preputial space and reduces this local immune activation.

If this hypothesis is true, then what causes the pro-inflammatory immune milieu that is seen in foreskin tissues adjacent to the sub-preputial space? Emerging evidence suggests that both co-infections and the local polymicrobial community (the penile microbiome) play a key role. Circumcision reduces the incidence of viral co-infections, particularly human papilloma virus (HPV) and herpes simplex virus type 2 (HSV-2) [1]. HSV-2 infection increases HIV risk, both in individuals with ulcerative disease where there is long-lasting tissue infiltration of activated CD4 T-cells [33], and also in asymptomatic men where it induces inflammatory foci and the selective foreskin recruitment of CCR5+ CD4 T-cells [34, 35]. HPV is also associated with HIV acquisition, perhaps because host HPV clearance is associated with an increased density of dendritic cells in both the foreskin [36] and female genital tract [37, 38]. However, the protective effect of circumcision against HIV infection is far greater than can be explained by a reduction in these viral co-infections alone [39]; another important mechanism by which circumcision may reduce penile inflammation is through the dramatic alterations that it induces in the penile microbiome [40, 41].

Over 42 distinct bacterial families can be found in the sub-preputial space of uncircumcised men, and gram-negative anaerobic genera that are associated with bacterial vaginosis in women are common [41]. For instance, *Prevotella* spp. were present in the foreskin prepuce of 87% of uncircumcised Ugandan men, where they constituted over 20% of the total bacterial load, and their abundance was increased (by 4.6 × 10^5^ 16S rRNA gene copies per swab) in the foreskin of men whose female sexual partner had BV [42]. Furthermore, there was a strong association in uncircumcised Ugandan men between the presence of BV-associated anaerobes in the foreskin prepuce and subsequent HIV acquisition. For instance, preputial *Prevotella* spp. were associated with an increased risk of HIV acquisition (adjusted OR 1.63, 95% CI 1.23–2.26), and their density was almost tenfold higher in men who acquired HIV (1.5 × 10^8^ vs. 1.9 × 10^7^ rRNA gene copies per swab in controls) [43], which is very analogous to BV-associated increases in female HIV susceptibility [44, 45]. These anaerobes were observed in the absence of penile symptoms, but their abundance correlated strongly with preputial levels of IL-8 (P < 0.01) and with the simultaneous detection of multiple chemokines (OR 4.8, 95% CI 2.4–9.6) [43], strongly suggesting that preputial anaerobes induce a local inflammatory response. However, while their causal role in driving genital inflammation in women has been demonstrated through the dramatic immune alterations induced by BV therapy [46], the direction of causality in men has not been defined, and may require clinical trials to assess the immune impact of microbiome-directed interventions.

Circumcision reduces both the total bacterial load on the penis and also specifically reduces the relative abundance of these anaerobic genera associated with HIV acquisition [40, 41]. Significant penile microbiome changes are apparent within 6 months of circumcision [40], and anaerobes continue to decline significantly for at least 2 years post-operatively, mirroring the progressive declines seen in IL-8 levels [24]. While vaginal bacterial dysbiosis is accepted as a driver of vaginal inflammation [47, 48] and HIV acquisition [49], the concept that penile bacterial dysbiosis may also drive inflammation and HIV acquisition is new. Coupled with the observation that the genital microbiome is shared between sexual partners, these observations have important implications for HIV prevention, but it remains to be shown whether they can be translated into prevention methods that extend beyond male circumcision. Specifically, because substantial HIV risk remains after circumcision and many at-risk men in HIV endemic regions choose to remain uncircumcised [50], it will be important to assess whether HIV risk can be reduced through clinical interventions that target penile immunology and/or the penile microbiome, both in uncircumcised but also circumcised men. Furthermore, it will important for preclinical and early phase clinical trials of novel HIV prevention methods, including HIV vaccines that aim to induce mucosal immune responses, to define intervention impacts on both genital inflammation and the genital microbiome.

## Conclusions

In summary, we hypothesize that immune activation in foreskin tissues adjacent to the sub-preputial space facilitates HIV infection through a combination of epithelial barrier disruption, enhanced dendritic cell maturation, and the recruitment/activation of neutrophils and susceptible CD4 T-cell subsets such as Th17 cells, and that the genital microbiome may be an important driver of this immune activation. It might appear that understanding these mechanisms would be a moot point for men who undergo circumcision, but since circumcision is only 60% protective against HIV acquisition, it is possible—or even probable—that similar mechanisms underpin their residual HIV susceptibility, as well as HIV susceptibility in women. Therefore, new modalities to reduce genital immune activation and/or alter the genital microbiome, used alone or in combination with topical microbicides, may be of significant benefit in HIV prevention.

## Abbreviations

HIV: human immunodeficiency virus-1
STI: sexually transmitted infection
GUD: genital ulcer disease
HSV-2: herpes simplex virus type 2
HPV: human papilloma virus
SIV: simian immunodeficiency virus
MIG: monokine induced by interferon-gamma
IL-8: interleukin-8
Th: helper T cell
HESN: HIV exposed SeroNegative
BV: bacterial vaginosis
